# Imaging Hepatocellular Carcinoma With ^68^Ga-Citrate PET: First Clinical Experience

**DOI:** 10.1177/1536012117723256

**Published:** 2017-09-11

**Authors:** Carina Mari Aparici, Spencer C. Behr, Youngho Seo, R. Kate Kelley, Carlos Corvera, Kenneth T. Gao, Rahul Aggarwal, Michael J. Evans

**Affiliations:** 1 Department of Radiology and Biomedical Imaging, University of California, San Francisco, San Francisco, CA, USA; 2 Helen Diller Family Comprehensive Cancer Center, University of California, San Francisco, San Francisco, CA, USA; 3 Department of Surgery, University of California, San Francisco, San Francisco, CA, USA; 4 Department of Pharmaceutical Chemistry, University of California, San Francisco, San Francisco, CA, USA

**Keywords:** transferrin, transferrin receptor, hepatocellular carcinoma, molecular imaging

## Abstract

While cross-sectional imaging with computed tomography (CT) and magnetic resonance imaging is the primary method for diagnosing hepatocellular carcinoma (HCC), they provide little biological insight into this molecularly heterogeneous disease. Nuclear imaging tools that can detect molecular subsets of tumors could greatly improve diagnosis and management of HCC. To this end, we conducted a patient study to determine whether HCC can be resolved using ^68^Ga-citrate positron emission tomography (PET). One patient with recurrent HCC was injected with 300 MBq of ^68^Ga-citrate and imaged with PET/CT 249 minutes post injection. Four (28%) of 14 hepatic lesions were avid for ^68^Ga-citrate. One extrahepatic lesion was not PET avid. The average maximum standardized uptake value (SUV_max_) for the lesions was 7.2 (range: 6.2-8.4), while the SUV_max_ of the normal liver parenchyma was 4.7 and blood pool was 5.7. The avid lesions were not significantly larger than the quiescent lesions, and a prior contrast CT showed uniform enhancement among the lesions, suggesting that tumor signals are due to specific binding of the radiotracer to the transferrin receptor, rather than enhanced vascularity in the tumor microenvironment. Further studies are required in a larger patient cohort to verify the molecular basis of radiotracer uptake and the clinical utility of this tool.

## Introduction

Hepatocellular carcinoma (HCC) is the most common type of liver cancer and the third most common cause of cancer-related deaths.^1^ Asymptomatic progression and thus difficult early detection, as well as underdeveloped therapeutic strategies, contribute to the very high fatality of HCC. Moreover, recent large-scale tissue studies have suggested that HCC can be classified as molecular subtypes with discrete tumorigenic profiles.^2^ On this basis, there is an urgent need to improve diagnostic strategies to empower the best treatment planning for this heterogeneous and very fatal disease.

While cross-sectional imaging with computed tomography (CT) and magnetic resonance imaging is the primary method for diagnosing HCC, they provide little insight into tumor biology. We hypothesized that nuclear imaging tools capable of detecting tumors with actionable oncogenic drivers could greatly improve diagnosis and management of this highly fatal cancer. Consistent with this hypothesis, radiotracers such as 18F-fluorodeoxyglucose (^18^F-FDG) and 18F-fluorocholine have been found to be beneficial for the detection of HCC and the determination of tumor differentiation in situ. However, the molecular basis of HCC avidity for these radiotracers is not known, and they do not yet provide additional biological information about the tumor subtype that might guide clinical decision-making.^3,4^

As part of a larger research program to develop probes for nuclear imaging to study the activity of central oncogenes,^5^ we and others have shown in preclinical models and clinical biopsies that the transferrin receptor (TFRC) is upregulated in tumors with important oncogenic drivers like the transcription factor MYC and the cytosolic Ser/Thr kinase mammalian target of rapamycin (mTOR).^6-8^ Hepatocellular carcinoma frequently harbors MYC and mTOR hyperactivity. These data led us to conduct the first human study to test whether any HCC tumors detectable with positron emission tomography (PET) targeting TFRC. ^68^Ga(III) is an Fe (III) biomimetic and potently and selectively binds to apotransferrin in blood,^9^ forming ^68^Ga-transferrin. To establish whether HCC tumors harbor high avidity for radiolabeled transferrin, we opened a protocol at University of California, San Francisco (UCSF) testing whether HCC could be detected with ^68^Ga-citrate PET.

We recently reported the first guidelines for ^68^Ga-citrate PET detection of castration-resistant prostate cancer metastases.^10^ We found that >130 MBq (or 3.5 mCi) and acquisitions >180 minutes post injection were required for suitable image quality and tumor resolution. During this study, a visceral prostate cancer metastasis in the liver was also detected, showing that tumors can be resolved against the background of normal liver, which also expresses high levels of the TFRC.

## Methods

### Positron Emission Tomography/CT Technique

Informed consent was obtained prior to enrolling the patient for this study. The study was approved by the institutional review board at UCSF. ^68^Ga-citrate 300 MBq (8.11 mCi) was injected intravenously. After 249 minutes of injection, a PET/CT was performed on a Discovery VCT PET/CT scanner (GE Healthcare, Waukesha, Wisconsin) with an integrated PET and 64-multidetector CT scanner. First, a single-bed PET acquisition over the liver was obtained for 10 minutes, followed by whole-body PET from vertex to thighs over 8 bed stations. Each PET was acquired for 4 minutes per bed station. A low-dose CT was obtained for attenuation correction only. Positron emission tomography images were reconstructed in a transverse matrix of 128 × 128, using a postreconstruction filter (8 mm Full Width at Half Maximum Gaussian), 14 subsets and 6 iterations of an ordered-subsets expectation maximization algorithm.

Positron emission tomography images were reviewed on an Advantage Workstation (GE Healthcare) by a dually trained abdominal radiologist and nuclear medicine physician with over 10 years interpreting PET (S.C.B.) and prior experience interpreting ^68^Ga-citrate PET data. Maximum and average standardized uptake values (SUV_max_ and SUV_ave_) were determined in the uninvolved liver and left ventricle by drawing a circular region of interest (diameter = 1 cm). Hepatic lesions were considered positive if they were visually detectable above normal liver uptake of the radiotracer. For positive HCC lesions, SUV_max_ was determined by drawing a 3-dimensional volume of interest encompassing the entire lesion.

Since the CT portion of the PET/CT was performed as low-dose CT, a multiphase diagnostic CT of the abdomen and pelvis performed 1 month prior to ^68^Ga-citrate PET/CT was used to assess the number and size of the lesions.

## Results

A 27-year-old man with a history of Crohn disease and treated with azathioprine was consented for this study. Three years prior to this study, a left hepatic lobe lesion was found during a magnetic resonance enterography. A multiphase CT showed 2 masses in hepatic segment 4 with imaging features consistent with HCC, whereupon the patient was treated with partial left hepatectomy. The patient was found to have recurrent multifocal disease in the right hepatic lobe on subsequent examinations. The most recent multiphase CT performed 1 month prior to the ^68^Ga-citrate PET/CT showed persistent, multifocal disease in the residual left hepatic as well as an abdominal wall implant. At the time of PET/CT, the patient’s serum α-fetoprotein concentration was 5755 μg/L.

Following our guidelines developed for prostate cancer, we injected the patient with 300 MBq (or 8.11 mCi) of ^68^Ga-citrate intravenously. After 249 minutes, the patient was imaged with PET/CT over multiple bed stations to determine the biodistribution of ^68^Ga-citrate.

**Figure 1.**
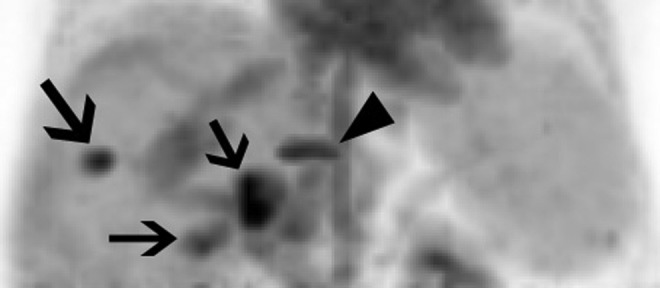
A maximum intensity projection showing multiple foci avid for ^68^Ga-transferrin. Tumor lesions are indicated with black arrows. The linear uptake corresponds to uptake from a vertebral body compression fracture (black arrow head).

**Figure 2.**
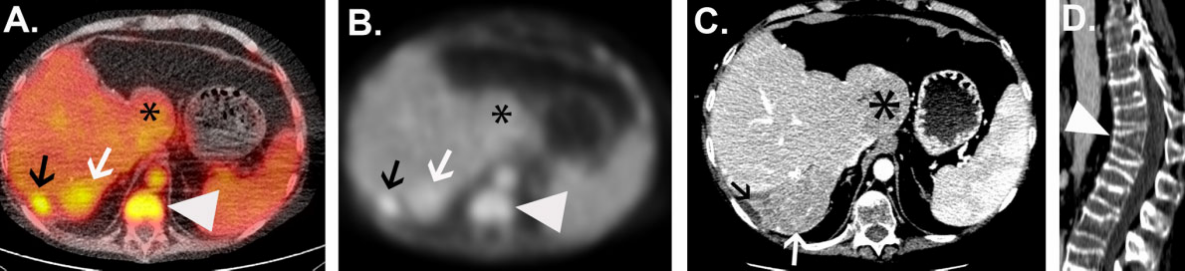
Representative images of ^68^Ga-transferrin uptake in tumor and normal structures. A, An axial PET/CT demonstrating variable uptake in 3 different hepatic masses. The most avid lesion (black arrow) corresponds to the 2.7 cm × 1.3 cm (SUV_max_ = 7.9) on the prior contrast-enhanced CT. A second lesion just medial to this (white arrow) is 5.7 cm × 5.7 cm, though had less radiotracer uptake (SUV_max_ = 6.2). A third lesion in the caudate lobe (*) measured 6.5 cm × 6.3 cm with uptake similar to uninvolved liver (SUV_max_ = 3.5). Focal uptake in the spine (white arrow head) corresponds to the vertebral body compression deformity seen on sagittal CT. B, The corresponding CT collected during the PET/CT acquisition. C, The contrast-enhanced CT collected 1 month prior to the PET/CT. D, A sagittal view of the CT showing the vertebral body compression. CT indicates computed tomography; PET, positron emission tomography; SUV_max_, maximum standardized uptake value.

A summary of the lesion size and SUV_max_ is outlined in Table 1. The average size of the lesions was 4.2 ± 1.8 cm (range: 1.7-6.9 cm). Four (28%) of 14 lesions were PET avid (Figure 1). The average size of the PET avid lesions was 4.7 ± 1.7 cm (range 2.7-6.5 cm). The average size of the quiescent lesions was 3.9 ± 1.9 (range: 1.7-6.9). The average SUV_max_ of the PET avid lesions was 7.5 ± 1.2 (range: 6.2.-8.9). The average SUV_max_ of the quiescent lesions was 3.2 ± 0.8 (range: 1.7-4.2; see Figure 2A-C). An anterior abdominal wall implant was the only site of extrahepatic disease. It measured 2.8 cm and was not PET avid (SUV_max_ = 2.7).

**Table 1.** Summary Table of liver lesions.

Segment Location	Size (cm)	SUV_max_	PET Avid
8	6.9	3.3	No
7	6.1	3.6	No
7	2.8	1.8	No
7	5.7	3.2	No
7	2.8	4.2	No
7	2.5	4.1	No
7	2.7	7.9	Yes
7	5.7	6.2	Yes
6	1.8	3.4	No
6	1.7	3.6	No
6	3.7	8.9	Yes
5	4.3	2.9	No
5	5.2	1.7	No
1	6.5	6.9	Yes

Abbreviations: PET, positron emission tomography; SUV_max_, maximum standardized uptake value.

Each of the PET avid lesions had SUV_max_ values exceeding those of blood pool, liver, and normal reference tissues. Blood pool SUV_max_ and SUV_ave_ were 5.7 and 3.6, respectively. The SUV_max_ and SUV_ave_ of the normal, uninvolved hepatic parenchyma were 4.3 and 2.7, respectively. The SUV_max_ and SUV_ave_ of the right paraspinal muscle were 0.6 and 0.4, while the SUV_max_ and SUV_ave_ of the right sacrum were 1.9 and 1.6, respectively. Focal uptake (SUV_max_ = 7.2) was seen in the T11 vertebral body compression deformity perhaps due to inflammation (Figure 2D).

## Discussion

In this study, we showed that HCC can be detected by administering a PET/CT after injection of ^68^Ga-citrate. Approximately 30% of lesions were avid for the radiotracer, which is not unexpected given the molecularly heterogeneous landscape of this disease. Tumor uptake of the radiotracer exceeded that of blood pool at 4 hours, demonstrating the rapid dissemination of transferrin into peripheral tissues. Moreover, despite the high expression of TFRC in normal hepatocytes, HCC lesions within the liver could be clearly resolved against the normal liver parenchyma. Lastly, 1 nonmalignant focus of radiotracer uptake was observed and readily distinguishable from malignancy by CT.

Although ^68^Ga-transferrin can accumulate in abscesses with abnormal vascularity,^11^ several lines of evidence show that uptake in the tumor microenvironment is due to specific receptor binding rather than the enhanced permeability and retention effect (EPR). First, the magnitude of tumor uptake exceeds blood pool, which itself is somewhat high, and the tumor uptake is above the range of what might be considered EPR.^12^ In preclinical models, ^89^Zr-albumin has been used to quantify signal due to EPR in subcutaneous xenografts, and the highest uptake observed was ∼5% ID/g.^12^ Second, the contrast enhancement observed on this patient’s staging CT was uniform across the 14 hepatic lesions, yet only 4 lesions were PET avid. If ^68^Ga-transferrin localization to the tumor were due to EPR, one could reasonably expect roughly equal accumulation of the radiotracer in all 14 hepatic lesions and values roughly equivalent to blood pool activity.

The relatively high blood pool activity observed at 4 hours post injection underscores that ^68^Ga(III) in serum exists bound within a large biomolecule and does not circulate in the free salt form. This is consistent with the long-standing pharmacological model that ^68^Ga(III) exclusively binds apotransferrin in serum, both owing to the presence of a high affinity metal binding site and the overall high abundance of apotransferrin in serum.^13^ Persistent blood pool activity also highlights the need for further refinement of the imaging protocol. We are currently increasing the injected dose beyond 300 MBq and imaging at later time points post injection to determine any improvement in signal to noise.

Whether there is a component of ^68^Ga-transferrin uptake in the tumor microenvironment due to infiltrating peripheral blood mononuclear cells is currently not understood. Future studies are required to acquire the tissue needed to address, in particular, to determine the specificity of ^68^Ga-transferrin for HCC versus sites of inflammation. That said, should the radiotracer biodistribution be attributable to leukocyte accumulation in the tumor microenvironment rather than tumor autonomous TFRC expression, the observation that the leukocytes only measurably accumulated in 4 of 14 hepatic lesions is thought-provoking and could provide the foundation for hypothesis-driven research on the mechanism of leukocyte tropism to tumors.

Because conventional radiographic response assessment is unreliable in HCC and some tumors may not be amenable to biopsy due to location or risk of tumor seeding, improved noninvasive imaging modalities are greatly needed for diagnosis as well as response assessment in HCC. Nuclear imaging tools, including ^18^F-FDG, ^18^F- or ^11^C-choline, ^11^C-acetate, and radiolabeled analogues of glutamine, have been studied in humans.^14-16^ The current body of data suggests that ^18^F-choline and glutamine derivatives like F18-(4S)-4-(3-[^18^F]fluoropropyl)-L-glutamate may prove most useful for measuring tumor burden regardless of tumor subtype or differentiation. Developing a radiotracer that uniquely targets a molecular subtype of HCC could synergize well with these technologies. Future studies in a larger patient cohort are required to verify with the molecular analysis of tissue biopsies the biological basis of ^68^Ga-transferrin uptake. Following the example of previous radiomic studies in the field,^8^ we are optimistic that the biological drivers of transferrin uptake can be defined by comparing the differences in gene expression patterns among PET avid and quiescent HCC lesions. Validating which genes are responsible for the PET avid versus quiescent phenotype can be further tested in preclinical cancer models.
